# Influence of Long-Term Storage and UV Light Exposure on Characteristics of Polyurethane Foams for Cryogenic Insulation

**DOI:** 10.3390/ma16227071

**Published:** 2023-11-07

**Authors:** Beatrise Sture, Vladimir Yakushin, Laima Vevere, Ugis Cabulis

**Affiliations:** Polymer Laboratory, Latvian State Institute of Wood Chemistry, Dzerbenes Street 27, LV-1006 Riga, Latvia; beatrise.sture@kki.lv (B.S.); vladimir.yakushin@kki.lv (V.Y.); laima.vevere@kki.lv (L.V.)

**Keywords:** rigid polyurethane foams, cryogenic insulation, photodegradation, artificial ageing

## Abstract

Rigid polyurethane (PUR) foams have been the most effective insulation material used in space launchers since the beginning of cryogenic fuel use, due to their outstanding thermal and mechanical properties. In this study, spray-applied PUR foams using different ratios of amine-based catalysts were produced. Due to climate change, several restrictions have been made regarding the usage of blowing agents used for PUR foam production. Lately, hydrofluoroolefins (HFOs) have been suggested as an alternative for PUR foam production due to their low global warming potential (GWP) and ozone depletion potential (ODP), replacing the hydrofluorocarbons (HFCs) so far used. This change in blowing agents naturally altered the usage of catalysts. Reactive amine-based catalysts are less hazardous because of their low volatility and ability to react successfully with isocyanate or polyols. Spray-applied PUR foams with a potential application for cryogenic insulation were produced and tested for long-term storage, analyzing parameters such as the pH value of polyol composition, foaming kinetics (t_rise_, t_cream_), etc. Athermal analysis (TG, DSC) was also applied to developed materials, as well as artificial ageing by exposing samples to UV light. It was discovered that PUR foams obtained using reactive amine-based catalysts, such as Polycat 203 and 218, have a higher integral heat capacity, but polyol mixtures containing these catalysts cannot exceed a storage time of more than 4 months. It was also observed from artificial ageing tests of PUR cryogenic insulation by exposure to UV light that the thickness of the degraded layer reached 0.8 mm (after 1000 h), but no significant destruction of cellular structure deeper in the material was observed.

## 1. Introduction

Rigid polyurethane (PUR) foams have been used as an insulation material for cryogenic facilities for decades. Their low thermal conductivity, dimensional stability, and low density have made them a really promising cryogenic insulation material for the transportation and storage of liquid gases (for example, nitrogen, hydrogen, or natural gas), where efficient insulation is paramount for maintaining the stability and integrity of systems operating at extremely low temperatures [1,2,3,4]. Due to the rising demand for better insulation solutions in sectors including energy (e.g., refrigerators and freezers), aerospace, construction, and several other applications, researchers and engineers have been working nonstop to improve the performance of PUR foams over the years [5,6,7]. For space launches that use liquefied oxygen (LOX) and liquefied hydrogen (LH_2_) as propellants, cryogenic insulation is crucial. The fundamental specifications for insulation materials are that they should be lightweight, have good thermal insulation, particularly at low temperatures, and be non-flammable, despite the fact that some of these traits are quite contradictory. The material’s resistance to cryo-pumping effects and UV stability are two further crucial properties for external thermal insulation. Since cryogenic propellants are used by the top space contributors, space research centers in the United States [8], China [9], and the European Union [10,11] are conducting extensive research in the subject of cryogenics.

Commercially, PUR foams are obtained from petrochemical components, but several investigations have also been conducted towards the usage of renewable resources, more specifically—the usage of bio-polyols. An analysis of PUR foams obtained with bio-polyols showed that the characteristics of the material did not worsen in cryogenic temperatures (although the improvement was rather unsignificant), and that they can be used as an alternative for petrochemically based polyols, therefore increasing the usage of renewable materials [12]. Brenes-Granados et al. [13] studied PUR foams using bio-polyols and amine-based catalysts (for example, Polycat 5 which is also used in this study), and the results showed an enhanced stability at high temperatures and lower water absorption compared to commercially produced PUR foam. This can be explained by smaller cell size, which results in a more even distribution of mechanical stresses, suitable for use in low temperatures. Lower water absorption also gives an advantage for cryogenic moisture uptake, which is a significant characteristic for cryogenic insulation foams [9,14]. Also, extensive studies of PUR composites reinforced with bio-based fillers have been conducted, for example, using hemp powder and silica particles [15]. It was discovered that the addition of both micro- and nanosized phenolated and pure hemp fillers and silica can increase the compressive modulus and strength of PUR foams by at least 20% compared to pure commercially produced PUR foams, which could be promising for cryogenic insulation materials (in that case, more extensive research, particularly in cryogenic temperatures, must be conducted) [15]. Furthermore, it was also found that the addition of silica particles reduced the thermal degradation of PUR foam composite.

In some studies, PUR foams reinforced with carbon nanotubes and glass fibers have been investigated in cryogenic temperatures, which showed that reinforcement with glass fibers delivers an increased compressive and tensile strength [16]; meanwhile, carbon nanotube-reinforced PUR tends to be more brittle than PUR without filler [17]. It has also been observed that PUR foams obtained from rapeseed oil polyols and using only water as a blowing agent have a higher compression strength in cryogenic temperatures than commercially produced PUR foams [18].

For PUR foam production, several catalysts are used, which are responsible for the foam’s blowing (e.g., pentamethyldiethyltriamine Polycat 5) and curing (e.g., dicyclohexylmethylamine Polycat 12). Unfortunately, the majority of these catalysts are hazardous to the environment and to laboratory staff working with them [19]; therefore, novel, less-volatile catalysts are being incorporated into PUR foam production [20]. The same concerns regarding the negative impact on the environment goes for blowing agents used in PUR foam production, e.g., hydrochlorofluorocarbon HCFC-141b and hydrofluorocarbon HFC-245fa [21]; therefore, more blowing agents with low global warming potential (GWP) and ozone depletion potential (ODP) are being used. Lately, hydrofluoroolefins (HFOs) or so-called 4th generation blowing agents are used for PUR production, replacing previously used hydrofluorocarbons (HFCs) [22,23]. These 4th generation blowing agents not only reduce their impact on the environment by lowering greenhouse gas emissions, but also make it possible to modify the microstructure of the foam to provide improved thermal insulation properties. Concurrently, due to these changes, the reactive amine-based catalysts compatible with blowing agents, e.g., Polycat 30, have to be tracked down, enabling the refinement of mechanical strength and thermal conductivity [22].

As mentioned previously, catalysts chosen for PUR foam development are obliged to assist in the foam’s blowing and curing, e.g., dimethylaminopropyldipropanolamine PC CAT^®^ NP 10 or PC CAT^®^ NP 88 (tertiary amine blend). Studies have shown that blowing can usually be achieved by adding an amine-based catalyst, meanwhile curing requires metal–organic catalysts. Amine-based catalysts, more specifically, tertiary amine catalysts, have the ability to induce both isocyanate-hydroxyl and isocyanate-water reactions [20]. The usage of commercially available amine-based catalysts has also shown promising results regarding the long-term storage of polyol mixtures [24]. For the curing/gelling process of PUR foam producing, tin-based catalysts were typically chosen, but recently, bismuth-based catalysts are being used due to their comparatively lower negative impact on the environment. Replacing tin and other heavy metal (for example, mercury)-based catalysts with bismuth-based catalysts has shown successful results [20].

PUR foams are widely used for liquid natural gas (LNG) transportation due to their ability to ensure minimal heat transfer and long-term durability, besides rather low maintenance costs. If PUR foam is being used for liquefied gas transportation and storage, the effect of the surrounding environment on PUR foam’s characteristics also must be considered. Therefore, in addition to compositional advancements, the manipulation of foam properties through artificial ageing processes has gained extensive attention [25,26]. Ultraviolet (UV) light exposure has emerged as a versatile method for inducing controlled ageing effects in PUR foams. Exposing materials to UV light allows researchers to mimic the natural degradation processes that PUR foams might encounter over time, thereby providing insights into long-term performance and durability. In addition to contributing to the knowledge of structural changes and degradation mechanisms inside PUR foams, artificial ageing via UV light exposure also directs formulation optimization for increased service life under challenging cryogenic conditions.

Photodegradation on PUR foams has been studied [26]; it was found that PUR foams oxidate in UV light, causing degradation, although damage was smaller in foams containing aromatic structures. It has been observed that, during exposure to UV light, photooxidation and consecutive hydrolysis occur, which result in the formation of alcohols, acids, and amines, therefore modifying sample’s structure [27]. Azevedo et al. [25] obtained PUR samples from castor oil-based polyol and showed an increased hardness at the near surface. Although this increased hardness of PUR foams is rather a positive characteristic, samples subjected to UV light can be more fragile and overall mechanical properties can decrease. Of course, the addition of photostabilizers increases PUR foams’ stability to UV light [28].

This study supplements our previous research article [29], where PUR foam was developed using a fourth generation blowing agent and reactive catalysts, and the physical–mechanical characteristics of the material were studied in both cryogenic and ambient temperatures. In this study, however, foaming parameters, alongside the pH value of the polyol mixture used for spray-foam PUR production, are studied during long-term storage. As well, the artificial ageing of PUR samples using exposure to UV light is investigated.

## 2. Materials and Methods

### 2.1. Materials

Several polyether and polyester polyols Lupranol 3300, Lupranol 3508/1, and Lupraphen 1901/1 were used for PUR foam production and were purchased from BASF (Ludwigshafen, Germany). Also, for PUR foam production diehtylene glycol, technical grade, from Sigma-Aldrich (Steinheim, Germany), IXOL B 251 (Solvay Fluor, Hanover, Germany) and flame-retardant tris-(1-chloro-2-propyl) phosphate (TCPP) from Albermarle (Louvain-la Neuve, Belgium) were used. As surfactant, Silicone L-6915LV from Momentive Performance Materials (Leverkusen, Germany) was used for preparation. In various combinations catalysts bismuth-based Dabco MB20 and amine-based Polycat 5, Polycat 203, and Polycat 218 (Evonik GmbH, Essen, Germany) were used for PUR foam production. As an isocyanate component of PUR foam formulations, polymeric 4,4-methylene diphenyl isocyanate (pMDI) Desmodur^®^ 44V20L from Covestro AG (Leverkusen, Germany) was used with an NCO group content of 31.5% and an average functionality of 2.7. For production of PUR foams, physical blowing agent HFO-1233zd-E (known by trade name Solstice^®^ LBA) from Honeywell Fluorine Products Europe B.V. (Weert, The Netherlands) was used. Water in polyols was used as chemical blowing agent (no water was added in PUR foam production).

### 2.2. Methods

#### 2.2.1. Preparation of PUR Foam Samples

For characterization of polyol mixture stability, standard PUR foam cup-test was carried out, using Foam Qualification System FOAMAT 285. Cream time (t_cream_), rise time (t_rise_), gel time (t_gel_) and density of foam were controlled in this test. Variation of the polyol mixtures’ pH during long-term storage was also monitored.

For the spraying of foam panels, a high-pressure GlasCraft MH VR dispensing system and a spray gun (Probler P2 Elite) were used. Both components—polyol mixture and isocyanate—were heated till 40 °C (heating was performed by spray system itself and was done in order to increase flowability of components), and the pressure of spraying components was 120–140 bar. PUR foam was sprayed on aluminum sheet covered with a release agent and thickness of spray-applied panels was 50–60 mm.

Formulations of PUR foams are listed in Table 1 (sample codes are the same as in our previous study [29]).

For normal execution of cup-test, concentration of amine catalyst in polyol mixtures had been halved compared to real spraying composition. It was necessary so as to have enough time to install cup with mixed A and B components in right place for foaming parameters measurements after component mixing. Mixing time of components was 2 s. After preparation, all polyol mixtures were divided in required number of portions. Every portion was stored in hermetic vessel at temperature 18–20 °C. Vessel was opened only once before foaming and pH tests. For pH value measurements, pH-meter ADRONA AM1605 (Adrona, Riga, Latvia) was used (pH range from 0 to 14, accuracy ± 0.01).

#### 2.2.2. Thermal Stability Tests

The thermal stability of the PUR foams was studied via thermo-gravimetric analysis TGA under nitrogen atmosphere using Thermogravimetric Analyzer (Discovery, TA Instruments, New Castle, DE, USA). The crushed foam samples (about 5–6 mg) were heated in platinum crucibles from room temperature up to 700 °C at a heating rate of 10 °C/min and a gas flow rate of 20 cm^3^/min.

The thermal transitions in the selected PUR foams were studied via differential scanning calorimetry DSC measurements using DSC 823e (Mettler Toledo, Columbus, OH, USA). The crushed foam samples (about 5 mg) were heated in aluminum crucibles from 25 °C up to 180 °C at a heating rate of 10 °C/min in a nitrogen atmosphere.

#### 2.2.3. Artificial Ageing by Exposing Samples to UV Light

UV stability of designed foams was tested using artificial ageing of foam samples in controlled environment using QUV weathering chamber (Q-Lab, Saarbrücken, Germany). A fluorescent lamp UVA-340 (295–420 nm, max 340 nm) with the intensity 0.89 W/m^2^ was used. Foam samples were held at 60 °C temperature and ambient pressure. The degradation experiment was conducted for 1000 h; six samples of each PUR foam composition were tested. Periodically, samples were photographed with a photo camera as well as tested by Portable Spectrophotometers CM-2500d (Konica Minolta, Tokyo, Japan). The thickness of degraded layer during UV irradiation was measured using Stereo Microscope Leica S9I with integrated 10 MP camera (Leica Microsystems, Wetzlar, Germany). For each composition, 10 measurements were obtained in both experiments.

The FTIR spectra analysis was carried out for the selected PUR foam samples before UV irradiation (initial) and for the samples after 1000 h exposure to UV irradiation (irradiated). The initial foam samples and surface layer of irradiated foam samples were sliced ~2 mm and tableted using press with 100 kPa. The FTIR spectra were recorded with Thermo Scientific Nicolet iS50 spectrometer (Waltham, MA, USA), scanning range—from 4000 to 450 cm^−1^, number of scans—64, resolution—4 cm^−1^.

## 3. Results and Discussion

The results of long-term storage of PUR foams will be presented in this section. As mentioned in our previous study [29], the spray-foamed PUR foams’ coefficient of thermal conductivity reached 15.4 mW/(m∙K).

### 3.1. The Effect of Long-Term Storage on PUR Foams’ Kinetic Parameters

**Figure 1:**
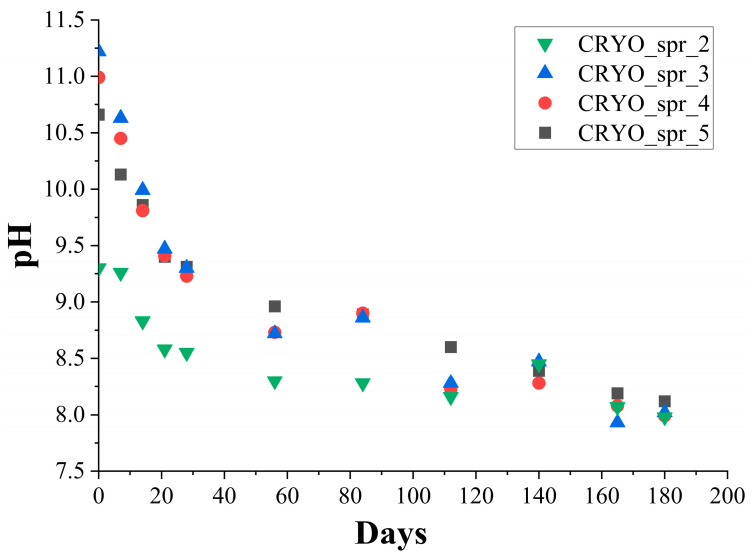
Change of pH level of polyol mixtures during long-term storage.

**Figure 2:**
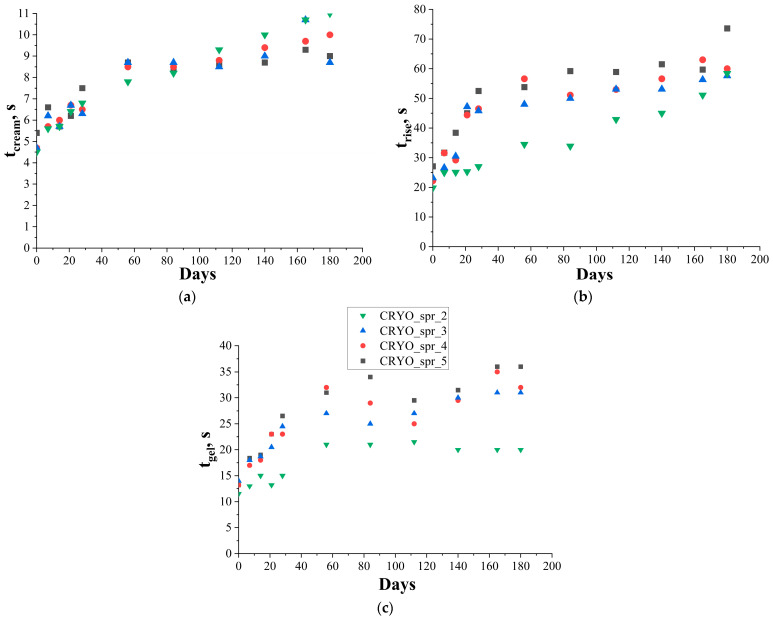
Change of PUR foam’s (**a**) t_cream_, (**b**) t_rise_ and (**c**) t_gel_ during long-term storage.

During the long-term storage of polyol mixtures, some side reactions between the halogen-contained blowing agent and flame retardant from one side, and the amine catalyst from the other side, take place. Due to such reactions, the activity of the catalyst and the pH of the polyol mixtures slowly decrease (see Figure 1). As a result of catalytic activity decrease, the foam rise profile of PUR foam composition was changed. As can be seen, during long-term storage, the cream time (see Figure 2a) and foam rise time (see Figure 2b) of PUR foam composition was increased. Consequently, the density of the foamed samples also increased.

Comparisons of the mentioned parameters’ variation during long-term storage of four polyol mixtures are presented in Figure 2. The cream time of the PUR foam compositions (see Figure 2a) during long-term storage of the polyol mixtures gradually increased from 4–5 s to 9–10 s. This happened most quickly in the first 2 months. The same picture was observed also for polyol mixtures’ pH decrease during storage in the first 2 months (see Figure 1). In the next 2 months, the process of pH and the cream time changes was slowed down somewhat, and then the rate of pH and the cream time changes were increased. The cream time of PUR foam composition with amine catalyst Polycat 5 increased the most.

The rise time of PUR foam compositions containing new amine catalysts Polycat 218 and Polycat 203 during long-term storage of polyol mixtures varied in the same manner as the cream time. In the first 2 months, the foam rise time of these compositions was increased relatively quickly from 22–23 s to 50 s. Then, after some plateaus in the last 2 months, the increase of the foam rise time continued. PUR foam composition with amine catalyst Polycat 5 had a lower initial foam rise time (20 s). During the long-term storage, the foam rise time of this composition was increased continuously.

**Figure 3:**
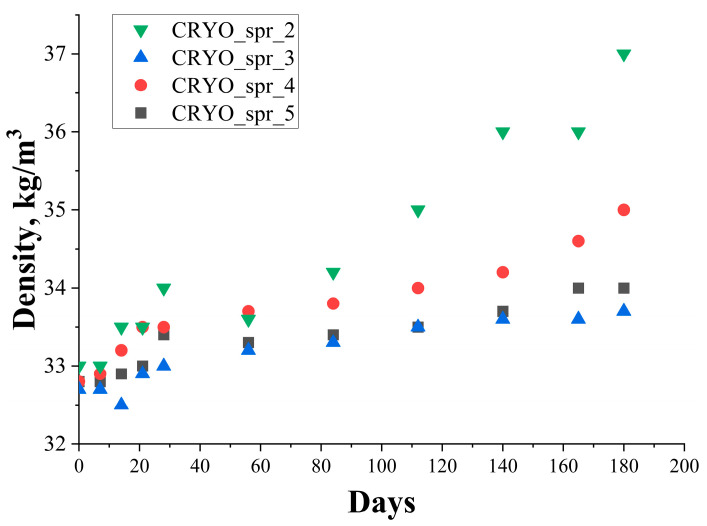
Change of PUR foam’s density during long-term storage.

Despite the fact that this composition with amine catalyst Polycat 5 had the shortest foam rise time, the density of this foam composition after long-term storage was higher (>35 kg/m^3^) than the density of PUR foam composition with new, especially designed for new age of blowing agent catalysts Polycat 218 and Polycat 203 (see Figure 3). The density of PUR foams with amine catalysts Polycat 218 and 203 was lower (<35 kg/m^3^) and some increase in the density was observed only after 4 months of polyol mixture storage. A significant increase in the density of PUR foam with amine catalyst Polycat 5 started after 3 months of polyol mixture storage.

Based on all the data, the maximum long-term storage time of polyol mixtures with amine catalyst Polycat 218 and Polycat 203 did not exceed 4 months. After four months of polyol mixture storage, the pH of the polyol mixture began to decrease significantly. As a result of this, the cream time, foam rise time, and the density of PUR foam also began to increase considerably.

### 3.2. PUR Foam’s Thermal Stability Tests

The physical properties and stability of the four best, previously mentioned spraying PUR foams with Solstice^®^ LBA (Honeywell, Phoenix, AZ, USA) and density about 35 kg/m^3^ were studied using thermogravimetric analysis (TGA), differential scanning calorimetry (DSC), and ultraviolet irradiation (UV) tests. All samples for tests were cut from the core of sprayed foam panels. The density of all samples varied from 34.1 to 35.0 kg/m^3^.

**Figure 4:**
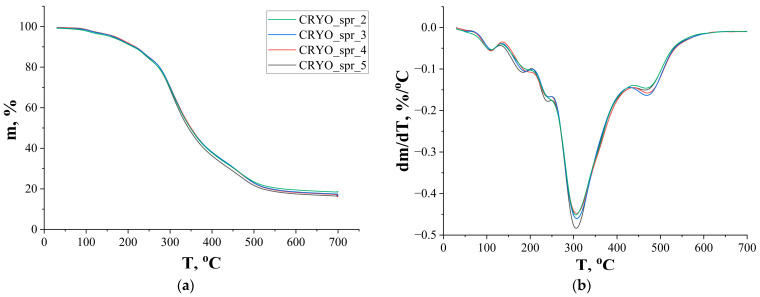
TG (**a**) and DTG (**b**) curves of PUR foams.

TG and DTG curves of the four selected PUR foams are presented in Figure 4a,b, respectively. Data from these curves are listed in Table 2. All PUR foams had a temperature of maximum rate of mass loss (T_max_) about 300–310 °C. This temperature can be considered as the temperature of PUR foam decomposition. The temperature of decomposition, as well as the temperatures of 5% and 10% of mass loss (T_5%_ and T_10%_), of PUR foams with amine catalyst Polycat 218 were higher than the relevant temperatures of PUR foam with amine catalyst Polycat 5 and foam with a combination of catalysts Polycat 218 and Polycat 203. Xu et al. obtained a slightly higher T_max_ temperature for his PU foams, but he also used higher TCPP content-20 pbw [30]. Char residue values at the temperatures of maximum rate of mass loss and at 500 °C (m_max_ and m_500_) of foam with a combination of catalysts Polycat 218 and Polycat 203 were higher than for other foams. But this foam had a peak of decomposition at 585 °C, and after this stage of thermal decomposition, foam with a combination of amine catalysts had minimal values of char residue at 700 °C (m_700_). This is probably due to the lower thermal stability of the compounds of this reactive catalyst (Polycat 203) with other elements of the polyurethane chemical structure.

**Figure 5:**
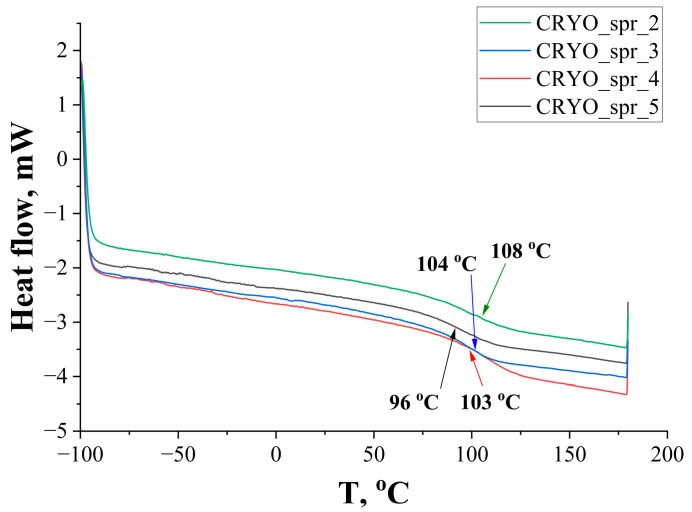
DSC curves of PUR foams.

DSC curves of selected PUR foams are presented in Figure 5. Tested PUR foams have a glass transition temperature between 96–108 °C. Typically, glass transition temperature increase is related to a higher crosslinking density. Polyol structure and functionality have the greatest impact on crosslinking density, but catalysts have some influence as well. Boquillon and Frigant described this in their research [31]. As CRYO_spr_2 has the highest glass transition temperature, Polycat 5 forms a higher crosslinking density than the rest of used amine catalysts. Partial replacement of Polycat 218 with Polycat 203 leads to a lower crosslinking density, as CRYO_spr_5 has a lower glass transition temperature than CRYO_spr_3 and CRYO_spr_4.

### 3.3. Artificial Ageing by Exposing Samples to UV Light

#### 3.3.1. Change of Color

**Figure 6:**
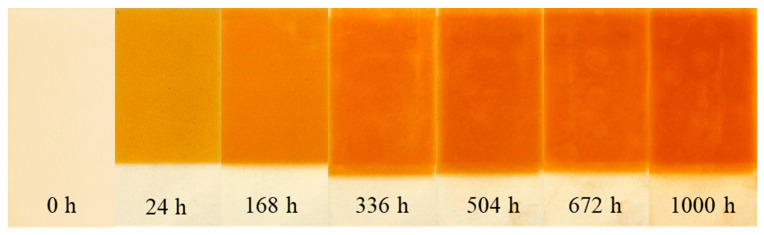
Color changes of PUR foam sample CRYO_spr_4 during artificial ageing via exposure to UV light.

During UV irradiation, the aromatic structures are oxidized in the central methylene group, leading to highly conjugated quinone products [32,33,34]. The accumulation of quinone products due to the chain scission of the PUR macromolecules induces the formation of colored products. The results of color changes have been documented with photographic evidence, and an example of sample CRYO_spr_4 is presented in Figure 6 (changes of other PUR samples are presented in Appendix A).

The color difference calculation was performed based on the CIE 1976 (L*a*b*) color difference equation:

(1) $$\Delta E^{*}{}_{a,b}=\sqrt{{\left(L_{2}^{*}-L_{1}^{*}\right)}^{2}+{\left(a_{2}^{*}-a_{1}^{*}\right)}^{2}+{\left(b_{2}^{*}-b_{1}^{*}\right)}^{2}}$$

The lightness, L*, represents the darkest black at L* = 0, and the brightest white at L* = 100. The color channels, a* and b*, will represent true neutral gray values at a* = 0 and b* = 0. Subscript 1 means the values measured before UV exposure, while subscript 2 denotes the values measured after UV exposure. The (a_2_* − a_1_*) positive values describe a red shift but negative denote a green shift, (b_2_* − b_1_*) positive values characterize the yellow shift, while negative values describe the blue shift. For example, the results of measurements and calculations for PUR foam CRYO_spr_4 are summarized in Table 3.

According to the given data, during UV irradiation, the surface of PUR foam samples gradually became dark (L*(D65) decreased) due to a surface color shift in the red and yellow areas of the spectrum (a*(D65) and b*(D65) increased). Visually, color variations of all PUR samples during UV irradiation are presented in Appendix A.

**Figure 7:**
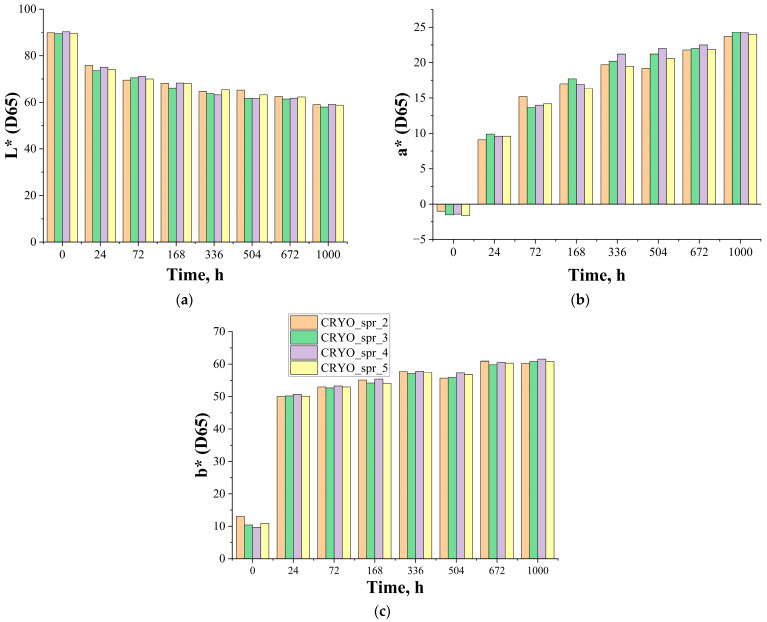
Change of (**a**) lightness L* (D65), (**b**) a* (D65), and (**c**) b* (D65) of PUR foam samples during artificial ageing via exposure to UV light.

**Figure 8:**
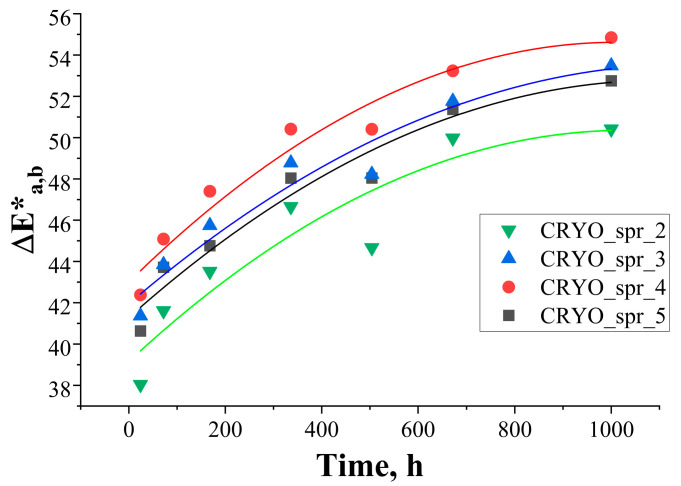
Change of color difference ΔE*_a,b_ of PUR foam samples during artificial ageing via exposure to UV light.

Comparisons of spectrophotometric results for all four PUR foam samples are presented in Figure 7 and Figure 8.

As can be seen, the effect of catalyst variation in PUR foam recipes on L*(D65) and b*(D65) parameters is insignificant. The effect of catalyst variation on a*(D65) parameters is bigger, and as a result of it, PUR foam CRYO_spr_2 has smaller color changes but PUR foam CRYO_spr_4 has larger color changes. Visually, the surfaces of these samples are browner (as can be observed in Appendix A).

#### 3.3.2. Thickness of Degraded Layer

**Figure 9:**
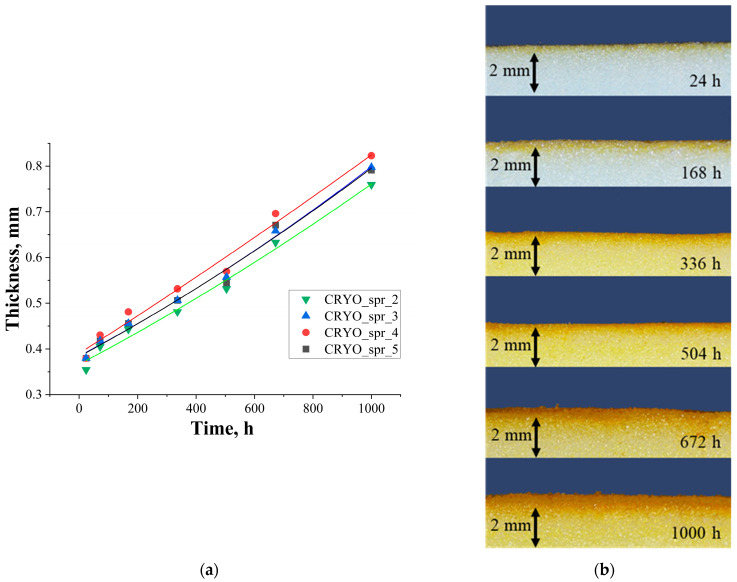
Thickness of PUR foams’ degraded layer during artificial ageing via exposure to UV light (**a**) and visual footage of PUR foam sample CRYO_spr_4 degraded layer during artificial ageing via exposure to UV light (**b**).

When measuring the depth of the foam degradation, the distance from the surface of the sample to the boundary of the transition of foam color from white to brown was taken as the foam degraded layer thickness. Typical photo images of UV-irradiated PUR foam samples’ cross-sections obtained with Stereo Microscope Leica S9I (Leica Microsystems, Wetzlar, Germany) are presented in Appendix A. Results of thickness measurements are presented in Figure 9a. A visual representation of the degraded layer of PUR foam sample CRYO_spr_4 is presented in Figure 9b. These figures show that the thickness of the degraded foam layer gradually increased during UV irradiation and reached 0.8 mm at 1000 h of irradiation, which is an improvement, considering that in [26] the thickness of the degraded layer after 1000 h reached over 2 mm. At the same time, significant destruction (for example, cracks further in the PUR foam’s depths which would not make it suitable for cryogenic insulation) of cellular structure was not observed. The effect of catalyst variation on the thickness of the degraded layer was higher for PUR foam CRYO_spr_3 and lower for PUR foam CRYO_spr_2.

#### 3.3.3. FTIR Analysis

**Figure 10:**
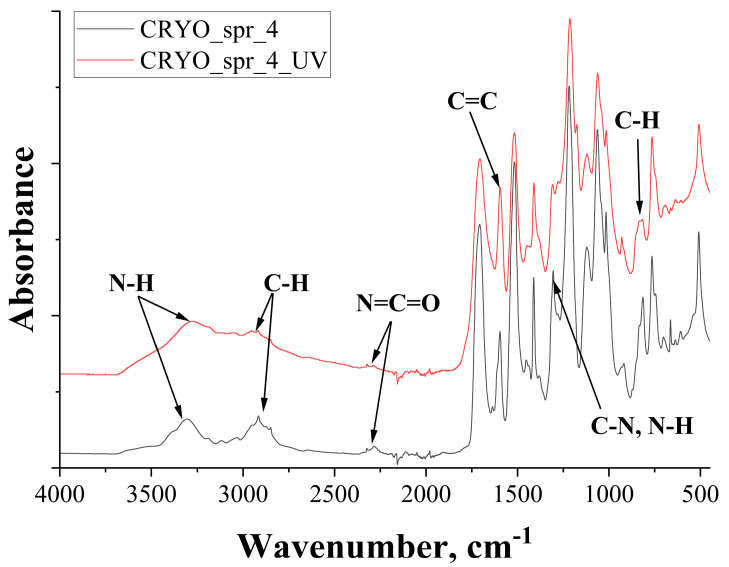
FTIR spectra of initial (CRYO_spr_4) and irradiated (CRYO_spr_4_UV) PUR foam samples.

Comparison of the four selected foams spectra showed that the effect of different amine catalysts on PUR foam spectra was negligible. The initial FTIR spectra between the four foams, as well as spectra between irradiated foams, were practically identical. The only observed difference was between the spectra of initial and irradiated foams. Therefore, for example, only the comparison of initial and irradiated PUR foam CRYO_spr_4 FTIR spectra is presented (see Figure 10).

Comparison of initial and irradiated PUR foam FTIR spectra showed that, due to UV degradation of PUR foam, the intensity of N-H stretching vibration at wavenumber 3292 cm^−1^ and C-H stretching vibration at wavenumber 2916 cm^−1^ peaks had widened and increased [35]. Also, a peak at 2290 cm^−1^ in CRYO_spr_4 spectra N=C=O stretching confirms that there is an excess of isocyanate groups in the polymer structure [35,36,37]. In CRYO_spr_4_UV spectra, it can be observed that this peak has decreased. A peak at 1600 cm^−1^, which indicates C=C stretching vibrations, can be conjoined with a C-H bending vibration peak at 810 cm^−1^ characterizing a 1,4-disubstituted aromatic ring [26]. Intensity of the peak at wavenumber 1596 cm^−1^, which indicated C=C stretching vibration in irradiated PUR foam due to the quinoid structure formation, also increased. The intensity of the peak at 1514 cm^−1^ which is relevant to Amide II (N-H bending and C-N stretching vibration) in the spectrum of irradiated PUR foam decreased [36]; at the same time, in the spectrum of irradiated PUR foam, additional peaks of Amide III (C-N stretching and N-H bending vibration) at 1309 cm^−1^ appeared.

## 4. Conclusions

According to the test data, the maximum long-term storage time of polyol mixtures with amine catalysts Polycat 218 and Polycat 203 cannot exceed 4 months. After this term, the polyol mixture pH begins to decrease significantly, and as a result, the cream time, foam rise time, gel time, and the density of PUR foams also begin to increase.

Each of the FTIR spectra for initial samples, as well as for each of the irradiated samples, are almost identical. However, certain differences were detected between each composition’s initial and irradiated samples: due to UV irradiation, the intensity of N-H stretching vibration and C-H stretching vibration peaks in all foam samples widened and increased; the intensity of C=C stretching vibration in irradiated samples also increased. The intensity of Amide II in the spectrum of irradiated PUR foam decreased, while at the same time additional peaks of Amide III appeared.

During UV irradiation, all PUR foams, due to the presence of an aromatic group in the polymer structure, became browner. PUR foam CRYO_spr_2 has lower color changes, but PUR foam CRYO_spr_4 has stronger color changes. The thickness of the degraded foam layer after 1000 h of UV irradiation reached a thickness of 0.8 mm; mainly, it was a change in foam color. At the same time, significant destruction of cellular structure was not observed.

Foams developed for cryogenic insulation were shown to be stable to UV radiation over a medium-term period (3–4 months). This means that they can be used without additional UV-protection in technical solutions with a short UV exposure time and where weight saving is very important, e.g., space launchers. In technical solutions exposed to many years of UV exposure, such as LNG tankers, developed PUR foams, like all other aromatic PUR materials, must be protected with a UV-protection layer.

## Figures and Tables

**Table 1:** Formulations of PUR foams.

	Sample	CRYO_spr_2	CRYO_spr_3	CRYO_spr_4	CRYO_spr_5
Polyols	Lupranol 3300	55			
	Lupranol 3508/1				
	Lupraphen 1901/1				
	Diethylene glycol	25			
	IXOL B 251	20			
Flame retardant	TCPP	15			
Surfactant	Silicone L-6815LV	1.5			
Blowing agents	Solstice^®^ LBA	45.0	45.0	45.0	41.4
	Water	0			
Catalysts	Dabco MB 20	0.15	0.15	0.2	0.2
	Polycat 218		3	3	2
	Polycat 203				1
	Polycat 5	3			
pMDI	Desmodur^®^ 44V20L	147–160			

**Table 2:** Thermal decomposition parameters of PUR foams.

PUR Foam	CRYO_spr_2	CRYO_spr_3	CRYO_spr_4	CRYO_spr_5
T_5%_ (°C)	158	166	169	165
T_10%_ (°C)	212	216	218	213
T_max_ (°C)	304	307	306	306
m_max_ (%)	68	67	68	66
m_500_ (%)	24	23	23	22
m_700_ (%)	18	17	17	16

**Table 3:** Variation of spectrophotometric results of PUR foam sample CRYO_spr_4 during UV irradiation.

Test Duration (h)	L*(D65)	a*(D65)	b*(D65)	ΔE*_a,b_
Initial	93.4	−1.8	11.0	0
24	78.5	8.3	49.6	42.6
72	74.8	12.4	52.8	47.9
168	68.0	18.7	56.1	55.7
336	63.4	21.9	61.7	63.5
504	61.0	22.9	61.0	64.5
672	56.6	26.1	59.8	67.2
1000	55.9	26.5	59.7	67.7

**Figure A1:**
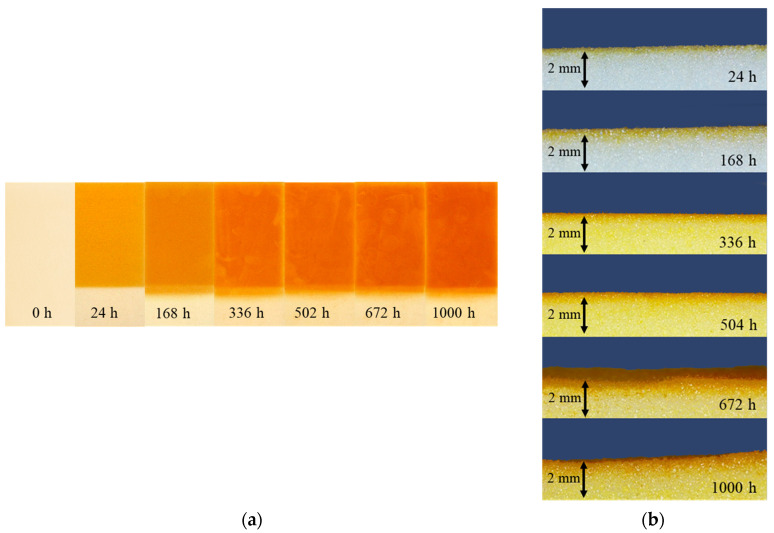
Color changes of PUR CRYO_spr_2 during artificial ageing via exposure to UV light (**a**) and visual footage of PUR foam sample CRYO_spr_2 degraded layer during artificial ageing via exposure to UV light (**b**).

**Figure A2:**
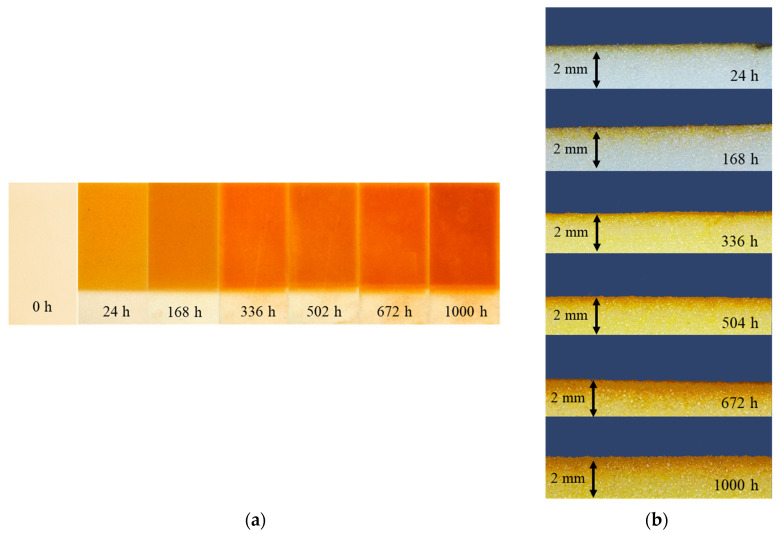
Color changes of PUR CRYO_spr_3 during artificial ageing via exposure to UV light (**a**) and visual footage of PUR foam sample CRYO_spr_3 degraded layer during artificial ageing via exposure to UV light (**b**).

**Figure A3:**
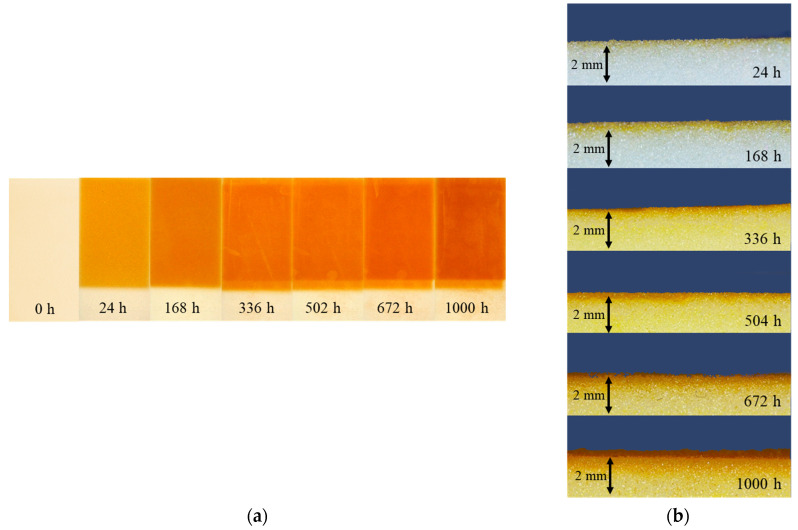
Color changes of PUR CRYO_spr_5 during artificial ageing via exposure to UV light (**a**) and visual footage of PUR foam sample CRYO_spr_5 degraded layer during artificial ageing via exposure to UV light (**b**).

## Data Availability

The data presented in this study are available on request from the corresponding author.
